# Whole‐genome sequencing of cell‐free DNA yields genome‐wide read distribution patterns to track tissue of origin in cancer patients

**DOI:** 10.1002/ctm2.177

**Published:** 2020-10-11

**Authors:** Han Liang, Fuqiang Li, Sitan Qiao, Xinlan Zhou, Guoyun Xie, Xin Zhao, Yongwei Zhang, Kui Wu

**Affiliations:** ^1^ BGI‐Shenzhen Shenzhen 518083 China; ^2^ Guangdong Provincial Key Laboratory of Human Disease Genomics Shenzhen Key Laboratory of Genomics BGI‐Shenzhen Shenzhen 518083 China

Dear Editor,

Somatic mosaicism is widespread among tissues and could indicate distinct origins for circulating cell‐free DNA (cfDNA) fragments, which are released by lytic cells in the tissues into the blood plasma.^1^, ^2^ We hypothesized that whole‐genome sequencing reads of different tissues could form type‐specific patterns in some regions as a result of somatic mosaicism. Whether this hypothesis could be used to construct a tissue‐of‐origin mapping model for cfDNA samples remains unknown.

To develop the model using read distribution patterns for tracking the tissue of origin of circulating tumor DNA (ctDNA), we first investigated the alignment patterns of sequencing reads from whole‐genome sequencing with the genomic DNA of 1545 tissue samples associated with 13 cancer types from the Pan‐Cancer Analysis of Whole Genomes (PCAWG) project of the International Cancer Genome Consortium (ICGC) and The Cancer Genome Atlas (TCGA).^3^, ^4^ Each cancer type included more than 60 donors (Table S1). Our technology includes the following four major steps (Figure 1, Supporting Information Materials and Methods): (a) Compute the number of reads (NR) aligned with each fixed‐width window. We divide each reference into a series of fixed‐width windows; the typical window length is 10 kbp, an empirically obtained value. For simplicity, we join all the chromosomes together (Y excluded) and obtain a chain of 257 973 windows (existing windows spanning two adjacent chromosomes). Then, we count the reads mapped inside each window for each sample to obtain the NR. (b) Search for frequently occurring read distribution patterns among the samples. This step attempts to summarize the landscape of samples of the same type based on frequently occurring patterns, where a pattern refers to the relationships among the windows according to their NRs (higher/equal/lower) (Figure 2a). We considered that only windows in close proximity would influence each other effectively. Notably, one pattern could include several windows if their relationships are common among samples. As an example, consider the pattern “D, C, A, B,” in which the letters indicate the window indexes. This pattern means that for many samples, the NR of window D is higher than that of window C, which is higher than that of window A, which is higher than that of window B. In other words, we rank windows by their NRs to describe their relationships simply (Figure 2b). (c) Extract type‐specific patterns from frequently occurring patterns. The previous step yields a large number of frequently occurring patterns by type. A pattern found often in one type of sample could also occur frequently in other types, and we need to extract those type‐specific patterns to construct a model. Here, we transform the Fisher's exact test *P*‐value to measure how “specific” a pattern is for one type compared to another. The transformed value is used as the weight of pattern, and more highly significant *P*‐values are always associated with higher weight values. Then, we extract patterns with weights above a calculated threshold. Obviously, when describing a type‐specific pattern, we must note from which type the pattern is frequently and from which type the pattern is rarely. (d) Identify the type of sample according to the type‐specific patterns. Two types of samples generate a paired type‐specific pattern sets that are extracted together. Here, a pattern “matches” a sample if the NR relationship among the windows described by the pattern is also valid for the sample. When we try to determine the possible type of a type‐unknown sample based on these two types, we observe how many patterns from each type‐specific pattern set match the sample. For each type, we accumulate the weights of the sample‐matching patterns to calculate a score. The type‐unknown sample will have two scores, one for each of the two types, and the type with the higher score would be considered the possible type of the sample. Obviously, if we have three or more types, we need to repeat step 3 for each pairwise combination of types and integrate all the results to obtain a final answer. After employing this method, we executed fivefold cross‐validation on the tissue samples and found that our model achieved an average accuracy of 81.38% (95% CI: 73.32‐89.45) (Figure 2c, Table S2).

**FIGURE 1:**
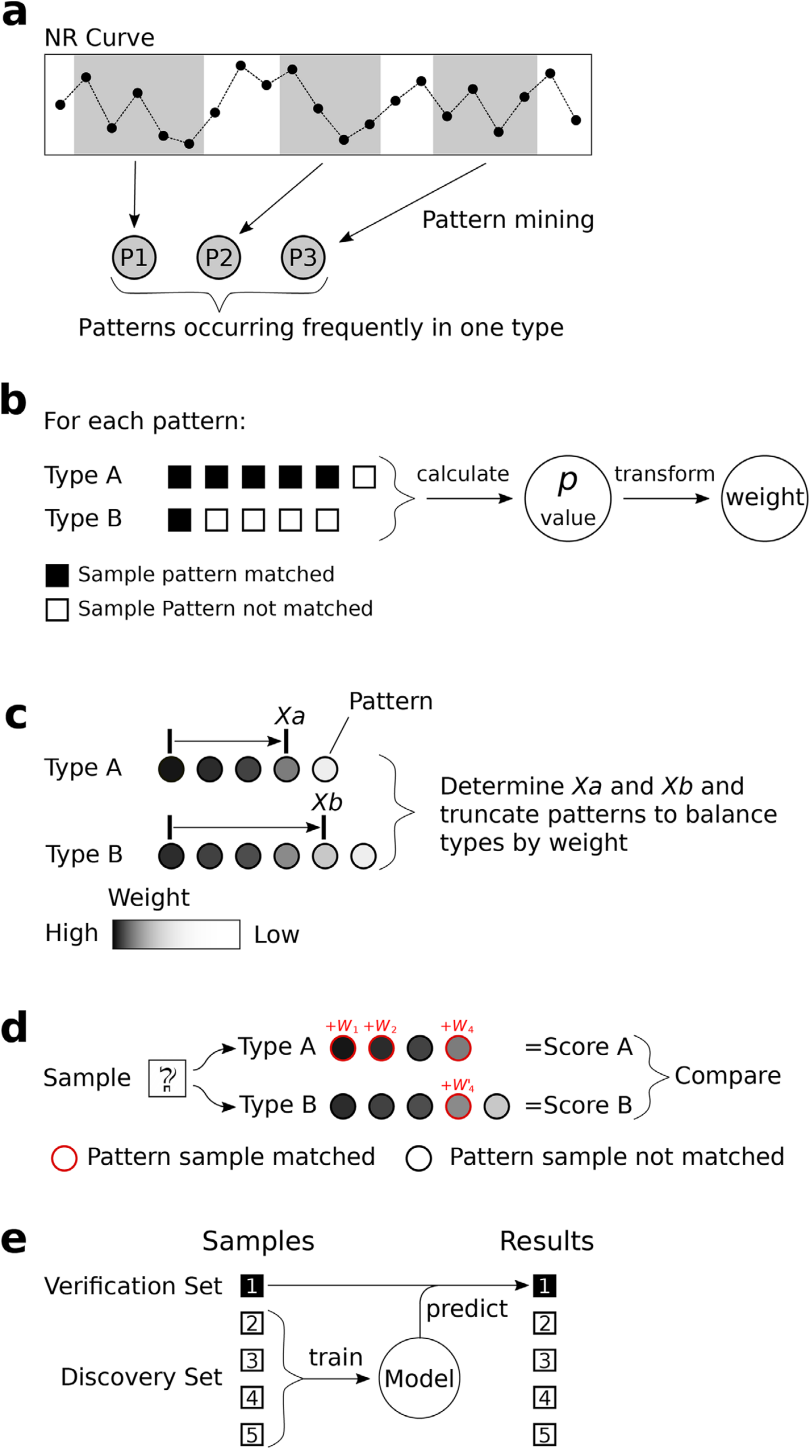
Schematic of model construction and prediction. **a**, Pattern mining. We searched for frequently occurring patterns in the NR curve for each type of sample. A pattern is considered frequent if its shape (the order of windows ranked by their NRs) is the same for most (e.g., 60%) of the samples’ NR curves. **b**, Type‐specific pattern extraction. For each pattern, we calculated the Fisher's exact test *P*‐value by checking how many samples were matched by the pattern in each type and then transformed the *P*‐value to derive the weight of pattern. We applied these operations to each pattern and retained patterns with a *P*‐value ≤ .01 as type‐specific patterns. **c**, Pattern balancing. We mined for frequently occurring patterns in type A samples, excluded the patterns found in type B samples and then repeated these steps by exchanging types A and B to obtain two type‐specific pattern sets. However, the resulting pattern sets were not necessarily balanced in weight. To resolve this problem, we used an optimization algorithm to derive *Xa* and *Xb* and retained the top *Xa* highest weight patterns for type A and the top *Xb* for type B to balance the weights. **d**, Sample prediction. To determine the type of a type‐unknown sample based on two type‐specific pattern sets, we checked whether the patterns matched the sample and summed the weights of the sample‐matched patterns for each type to obtain two scores, one per possible type. The type with the higher score would be considered the sample's possible type. **e**, We divided the tissue samples equally into five subsets and the plasma samples into 10 subsets. For each subset, we combined the remaining subsets into a discovery set to train a model and then predicted the original subset as the verification set. Then, we combined all the predicted results to analyze the performance of the model.

**FIGURE 2:**
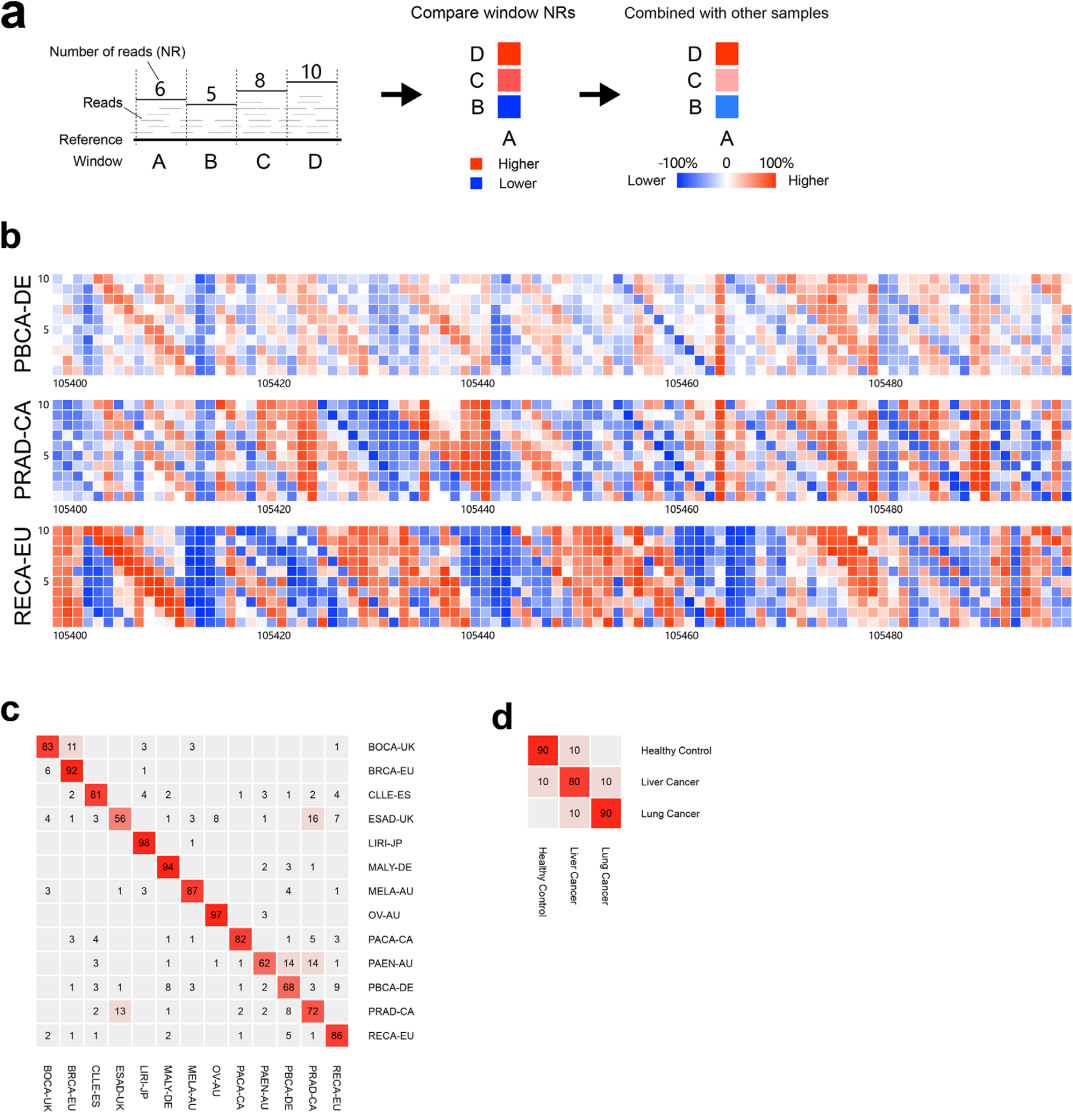
The read distribution pattern of a reference and predicted results. **a**, The generation of read distribution patterns. As shown, we divided the reference into four fixed‐width windows labeled A‐D, counted the number of reads (NR) mapped to each window, and performed pairwise comparisons of the windows based on their NRs to obtain their relationships (higher/equal/lower). In this example, the NR of window A is lower than that of window B, indicated by a blue square, and higher than those of C and D, indicated by red squares. To discuss the relationships between two windows in the context of multiple samples, we can assume that the two windows are labeled A and B. Then, we use a percentage to represent the difference between samples for which the NR of A is higher than that of B (n = Na) and those for which the NR of B is higher than that of A (n = Nb): the percentage value = (Na − Nb)/(Na + Nb) × 100%. **b**, The read distribution patterns of three types of tissue samples. We joined all the chromosomes (Y excluded) together and obtained a long chain of 257 973 windows (for hg19). The relationships among the windows ranked 105 400‐105 500, 100 windows in total, are shown for three cohorts. Each window was compared with its 10 downstream windows. The *x*‐axis represents the window index, the *y*‐axis represents the distance of the downstream windows from the current window, and the colors represent the percentages calculated with the method described in (**a**). **c**, The results of the tissue samples. This test involved 13 types of tissue samples, and the figure shows the integration of the fivefold cross‐validation results. The rows represent the different types of samples, the *y*‐axis labels (at the right) represent the real sample types, the columns represent the predicted results, and the *x*‐axis labels (at the bottom) represent the predicted types; the numbers inside the cells represent the percentages of samples predicted as each *x*‐axis label among the samples marked with each *y*‐axis label. **d**, The results of the cfDNA samples. This test involved three types of cfDNA samples, and the figure shows the integration of the 10‐fold cross‐validation results.

To evaluate our model's identification of tissues of origin for cfDNA samples, a total of 30 cfDNA samples from lung cancer and liver cancer patients and healthy controls were analyzed (Table S3). The cfDNA samples were sequenced on a BGISEQ‐500 with an average 3× depth of coverage. To explore the samples sufficiently, we performed 10‐fold cross‐validation on the cfDNA samples (rather than fivefold, which was used for the tissue samples, as 13 types of tissue samples had to be distinguished and the cost of 10‐fold cross‐validation would have been excessive). We found that our model achieved an average accuracy of 83.33% (95% CI: 68.99‐97.68) (Figure 2d, Tables S2 and S4). There were four erroneous results among the 30 samples: one healthy control sample was misidentified as liver cancer, one lung cancer sample was misidentified as liver cancer, and two liver cancer samples were misidentified, one as healthy control, and one as lung cancer. Notably, our model distinguished healthy control samples with high accuracy, which is very important in early tumor screening.

In summary, this study presents a new model that using the type‐specific reads distribution patterns caused by tissue‐specific somatic mosaicisms to track tissues of origin for ctDNA with high accuracy, suggesting the potential application of our model to early cancer detection and diagnosis. In the future, we will investigate more samples from different cancer types, inflammation, benign, and different centers to evaluated the robustness of the model.

## CONFLICT OF INTEREST

The authors declare no conflict of interest.

## ETHICS APPROVAL AND CONSENT TO PARTICIPATE

This study was approved by the by the Ethics Committee of Institutional Review Board of BGI. All samples were collected with written informed consent from adult participants, and minors’ informed consent was given by their guardians. All experiments were performed in accordance with relevant guidelines and regulations.

## CONSENT FOR PUBLICATION

All the authors consent for publication.

## AUTHOR CONTRIBUTIONS

Kui Wu, Yongwei Zhang, and Fuqiang Li conceived of the idea and supervised the work. Han Liang developed the model of tracking tissue of origin for ctDNA. Sitan Qiao and Guoyun Xie performed standard pipeline of sequencing data. Xinlan Zhou and Xin Zhao performed experiments of sequencing. Han Liang and Fuqiang Li wrote the manuscript. Kui Wu contributed to drafting and revising the manuscript.

## Supporting information

Supporting information

## Data Availability

Alignment files of 1545 tissue samples involving 13 cancer types from the Pan‐Cancer Analysis of Whole Genomes (PCAWG) project were analyzed on the Cancer Genome Collaboratory, an academic compute cloud resource that allows researchers to run complex analysis operations across large ICGC cancer genome data sets. The sequencing data of 30 cfDNA samples have been deposited into CNSA (CNGB Nucleotide Sequence Archive) of CNGBdb with accession number CNP0000680 (https://db.cngb.org/cnsa/). The up‐to‐date version of the analysis code, along with an up‐to‐date README, is available as a Github repository (https://github.com/lianghan-bgi/Reads-Distribution-Pattern). This manuscript has been released as a preprint at *bioRxiv*.^5^
