# Supplementary material for: Whole‐genome sequencing of cell‐free DNA yields genome‐wide read distribution patterns to track tissue of origin in cancer patients
Source: Clin Transl Med. 2020 Oct 11;10(6):e177. doi: 10.1002/ctm2.177 (PMC7548096; doi:10.1002/ctm2.177)
Supplement: Supplementary file 1 — Supporting information [file CTM2-10-e177-s001.docx]

**Supplementary Material for**

**Whole genome sequencing of cell-free DNA yields genome-wide read distribution patterns to track tissue of origin in cancer patients**

**Methods and Materials**

**Counting the read distribution on the reference.**

Here, we define sorted window indexes (SWIs), which refer to a series of indexes of windows placed on the reference. These indexes are sorted according to the number of reads mapped inside each window, or NR. In this paper, an SWI is considered a simplified read distribution.

First, we need to determine every sample’s SWI. This step is performed as follows:

1) Divide the reference into a series of fixed-width windows, and label those windows with their indexes. For simplicity, we join all the chromosomes together in numerical order (chromosomes 1-22 & X; Y excluded). The window length is typically set as 10 kbp, an empirical value, but sometimes, another value in the range of 5 kbp-50 kbp is used.

2) Count the reads mapped inside each window for each sample. The reads are mapped to the reference with a standard short read alignment method. When a read spans two windows, we consider the window in which most of its bases are located its mapped window.

3) Obtain the SWI for each sample by sorting the window indexes by NR.

4) Repeat steps 2 and 3 until all samples have been processed.

Each sample produces an SWI, and the SWIs gained from a sample set form an SWI set.

**Search for frequent distribution patterns among SWIs.**

A pattern is a series of order-sensitive numbers that refer to window indexes, e.g., (3, 1, 2); it is a miniature SWI. A pattern could contain a series of numbers too long to search directly, and we developed a splicing model to find such patterns:

If there are two patterns, one of which has a tail section identical to another pattern's head section, the operation of joining the former pattern with the latter pattern is called splicing.

As an example, consider two patterns, (1, 2) and (2, 3). The tail section of the former pattern is "2", which is the same as the latter pattern's head section. We take the former pattern (1, 2) and splice on the latter pattern's remaining tail section (3) to gain a new, longer pattern (1, 2, 3).

To reduce computational complexity, however, we splice only pairs of patterns whose indexes contain only one different element. For example, we could splice patterns (1, 2, 3) and (2, 3, 4) into a new pattern (1, 2, 3, 4), because the former and the latter contain only one different element each (“1” and “4”, respectively).

Splicing combines two shorter patterns to produce a longer pattern, and the shortest pattern contains only two indexes and is called an L2. We searched the L2s using the following formula:

L2(*d*, *n*)=(*i*, *j*) *i*ϵN, *j*ϵN, 1≤*i*≤*n*, 1≤*j*≤*n*, |*i*-*j*|≤*d*

where *n* is the maximum index among the windows, and *d* is the maximum distance between two indexes of L2. Based on empirical evidence, we set *d* as 40.

We are interested in frequently occurring patterns, and therefore, additional checks are required. To determine whether a pattern occurs frequently, we determine how many SWIs a pattern match. Here, we say a pattern “match” an SWI when the order of the windows contained in the pattern is the same as that in the SWI. For example, we say the pattern (3, 1, 2) matches the SWI (4, 3, 1, 2) because the order of “1”, ”2”, and ”3” in the pattern is the same as that in the SWI. Furthermore, the number of samples matched by a pattern must reach a given threshold to be considered a frequent pattern.

Then, we attempt to obtain all frequent patterns by splicing the patterns iteratively until no more frequent patterns are generated.

Finally, the frequent patterns obtained from a sample set form a frequent pattern set.

**Extracting type-specific patterns from frequent patterns**

A frequent pattern is type-specific if there is a significant difference in its coverage between two sample sets. First, we filter frequent patterns according to their coverage of two sample sets. Two pattern sets gained from two sample sets, after each is filtered with the other sample set, will generate two paired type-specific pattern sets. To measure the ability of a frequent pattern to distinguish two kinds of samples, we use the transformed Fisher’s exact test *p*-value as the pattern's weight. To calculate the *p*-value, we need to check how many samples in each sample set are matched by the pattern. The transformation formula is

$$f(x)=-log(max(1^{-100},x))$$

To assign a sample to one of the two types, we use the following formula:

$$\mathrm{score}\left( S,P \right)=\sum_{i=0}^{N} \left\{ \begin{aligned} weight\left( Pi \right), if order\left( P_{i},n_{i} \right)=order\left( S,n_{i} \right) \\ 0, otherwise \end{aligned} \right.$$

$$n_{i}=index(P_{i})\cap index(S)$$

where S is a SWI extracted from the type-unknown sample, P is one of the two paired type-specific pattern sets, N is the total number of patterns in P, weight(x) is the weight of pattern x, P_i_ is the *i*-th pattern in P, order(x, y) is the order vector of index set y in pattern/SWI x, and index(x) is the index set of pattern/SWI x.

Comparing the scores of the two type-specific pattern sets, the type corresponding to the higher score will be considered the possible type.

However, the differences between the weighted sums of two specific pattern sets can be very large, making one score always higher than another. Thus, we need to delete some patterns to balance the weights, which is done with the following formula:

$$\mathrm{balance}\left( X_{a},X_{b} | P_{a},P_{b},S_{a},S_{b} \right)=\underset{X_{a},X_{b}}{\mathrm{argmin}} p(co\left( S_{a},P_{a},{CX}_{a},P_{b},X_{b} \right),in\left( S_{a},P_{a},X_{a},P_{b},X_{b} \right),co\left( S_{b},P_{a},{CX}_{a},P_{b},X_{b} \right),in\left( S_{b},P_{a},X_{a},P_{b},X_{b} \right))$$

where X_a_ and X_b_ are the two factors to be determined. X_a_ is used as a length to retain the top *X_a_* highest-weight patterns of the type-specific pattern set P_a_, which was obtained from sample set S_a_; X_b_, P_b_ and S_b_ belong to the other sample type and have the same meanings as X_a_, P_a_ and S_a,_ respectively. p(*a*, *b*, *c*, *d*) is the *p*-value calculated using Fisher’s exact test with the factors *a*, *b*, *c*, *d*; co(*s*, *a*, *b*, *c*, *d*) is the number of samples predicted correctly in sample set *s* with pattern set *a* truncated with length *b* and pattern set *c* truncated with length *d*; in(*s*, *a*, *b*, *c*, *d*) is the number of samples predicted incorrectly with the same factors as function co.

To solve this formula, we use an iterative algorithm. First, we set X_a_ as a reasonable random value and find the best X_b_ in the given situation; then, we keep X_b_ unchanged and find the best X_a_. The end condition of this iterative process is stable values of X_a_ and X_b_.

Finally, we use X_a_ and X_b_ to truncate the two paired type-specific pattern sets.

**Identify a sample’s type according to type-specific patterns**

The method used to identify a sample’s type was introduced in the previous step for only two types. When trying to determine a sample’s type from more than two candidates, we need to repeat the previous step for every combination of two types. Obviously, for N types, there will be N(N-1)/2 combinations. In this situation, every repetition will provide a possible answer, and all these answers can be combined to determine a final prediction.

**cfDNA samples**

Whole blood of patients with lung and liver cancer and healthy volunteers was collected in EDTA tubes and stored at 4°C. Plasma was isolated immediately or within 24 hours by double centrifugation and stored at −80 °C. First, plasma and cellular components were separated by centrifugation at 1600g for 10 min at 4 °C. Then, the upper plasma was further centrifuged for 10min at 16,000g at 4 °C to remove any remaining cellular debris. The separated plasma was divided into 1 mL aliquots to avoid repeated freezing and thawing and stored at -80°C until use. Circulating cfDNA was extracted from 1 to 4ml of plasma using the QIAamp Circulating Nucleic Acid kit (catalog # 55114, Qiagen) according to manufacturer’s instructions. The concentration of cfDNA was quantified by the Qubit ﬂuorescence quantitative method with Qubit dsDNA HS Assay kit (Life Technologies, Carlsbad, CA). Next-generation sequencing (NGS) cfDNA library was prepared for whole genome sequencing (WGS). For each sample, quantified 10 ng of cfDNA was repaired to obtain a blunt end and modified at 3’end to get a dATP as a sticky end. Both ends of cfDNA were ligated with dTTP tailed adapter sequence. The ligation product was amplified for 10 cycles, and then subjected to single strand circularization process to obtaining a single strand circular DNA library through heat denaturation together with a special molecule which was reverse complemented to one special strand of the PCR product, ligation with DNA ligase and digestion with the exonuclease. All single strand circular DNA libraries were sequencing on the BGISEQ-500 platform with paired-end reads to obtain a average depth of 3X for the 30 cfDNA samples. The BWA algorithm was used to align the reads to the human reference genome hg19, and duplications were removed by Picard. This study was approved by the by the Ethics Committee of Institutional Review Board of BGI. All samples were collected with written informed consent from adult participants, and minors’ informed consent was given by their guardians. All experiments were performed in accordance with relevant guidelines and regulations.

**Supplementary tables:**

| **Supplementary Table 1. Details of Cancer Types from the PCAWG Project** | | |
| --- | --- | --- |
| **Cancer Type from the PCAWG Project** | **Cancer Type Abbreviation** | **Number of Donors** |
| Bone Cancer - United Kingdom | BOCA-UK | 76 |
| Breast ER+ and HER2- Cancer - European Union/United Kingdom | BRCA-EU | 79 |
| Chronic Lymphocytic Leukemia - Spain | CLLE-ES | 100 |
| Esophageal Adenocarcinoma - United Kingdom | ESAD-UK | 100 |
| Liver Cancer - Japan | LIRI-JP | 259 |
| Malignant Lymphoma - Germany | MALY-DE | 101 |
| Skin Cancer - Australia | MELA-AU | 70 |
| Ovarian Cancer - Australia | OV-AU | 73 |
| Pancreatic Cancer - Canada | PACA-CA | 148 |
| Pancreatic Cancer Endocrine Neoplasms - Australia | PAEN-AU | 69 |
| Pediatric Brain Cancer - Germany | PBCA-DE | 251 |
| Prostate Adenocarcinoma - Canada | PRAD-CA | 124 |
| Renal Cell Cancer - European Union/France | RECA-EU | 95 |

**Supplementary Table 2. The integrated accuracy of series verification sets**

| **Test** | **Cohort** | **Mean (%)** | **95% CI** |
| --- | --- | --- | --- |
| Tissue Samples | BOCA-UK | 83 | 75 - 90 |
|  | BRCA-EU | 92 | 84 - 101 |
|  | CLLE-ES | 81 | 61 - 101 |
|  | ESAD-UK | 56 | 33 - 79 |
|  | LIRI-JP | 98 | 96 - 100 |
|  | MALY-DE | 94 | 89 - 99 |
|  | MELA-AU | 87 | 80 - 95 |
|  | OV-AU | 97 | 90 - 105 |
|  | PACA-CA | 82 | 75 - 90 |
|  | PAEN-AU | 62 | 52 - 73 |
|  | PBCA-DE | 68 | 61 - 75 |
|  | PRAD-CA | 72 | 62 - 82 |
|  | RECA-EU | 86 | 72 – 101 |
| cfDNA Samples | Healthy | 90 | 67 - 113 |
|  | Liver Cancer | 80 | 50 - 110 |
|  | Lung Cancer | 90 | 67 - 113 |

**Supplementary Table 3. The summary clinical info of all participants offering cfDNA sample**

| **Group** | **ID** | **Gender** | **Age** | **Stage** | **Cancer** | **Smoking** | **Round*** |
| --- | --- | --- | --- | --- | --- | --- | --- |
| Healthy | 1 | Male | 30 | - | - | No | T3 |
|  | 2 | Male | 26 | - | - | No | T5 |
|  | 3 | Male | 30 | - | - | No | T1 |
|  | 4 | Male | 30 | - | - | No | T2 |
|  | 5 | Female | 27 | - | - | No | T6 |
|  | 6 | Female | 26 | - | - | No | T4 |
|  | 7 | Male | 26 | - | - | No | T7 |
|  | 8 | Female | 27 | - | - | No | T8 |
|  | 9 | Female | 31 | - | - | No | T9 |
|  | 10 | Female | 30 | - | - | No | T0 |
| Liver Cancer | 1 | Female | 39 | II | HCC | Unknown | T7 |
|  | 2 | Male | 70 | II | HCC | Unknown | T3 |
|  | 3 | Male | 62 | II | HCC | Unknown | T6 |
|  | 4 | Male | 59 | II | HCC | Unknown | T0 |
|  | 5 | Male | 67 | II-III | HCC | Unknown | T4 |
|  | 6 | Male | 62 | II | HCC | Unknown | T2 |
|  | 7 | Female | 65 | III | HCC | Unknown | T1 |
|  | 8 | Female | 80 | II | HCC | Unknown | T9 |
|  | 9 | Male | 82 | II | HCC | Unknown | T5 |
|  | 10 | Male | 58 | II-III | HCC | Unknown | T8 |
| Lung Cancer | 1 | Male | 67 | IV | NSCLC | No | T5 |
|  | 2 | Male | 52 | I | AD | No | T1 |
|  | 3 | Male | 63 | IV | SC | Yes | T6 |
|  | 4 | Male | 49 | IV | AD | Yes | T3 |
|  | 5** | Female | 55 | IV | AD | No | T0 |
|  | 6** |  |  |  |  |  | T7 |
|  | 7** |  |  |  |  |  | T9 |
|  | 8 | Male | 60 | IV | AD | Yes | T2 |
|  | 9 | Male | 51 | IV | AD | Yes | T8 |
|  | 10 | Female | 43 | IV | AD | No | T4 |

* Round column indicates which samples are used as verification set while the rest samples are used as discovery set in each round of test (T0~T9, 10 tests in total) in 10-fold validation, which is assigned randomly.

** Sample No.5/6/7 in lung cancer group both come from the same patient, they are extracted in 3 different time points: 1) before treatment (No.5); 2) before chemotherapy cycle 5 (No.6); 3) after chemotherapy cycle 6 (No.7).

**Supplementary Table 4. Nine patterns with the top 20 highest weights in each round test in the cfDNA sample based 10-fold validation.**

| **Pattern*** | **Group** | **Matched** | **Mismatched** |
| --- | --- | --- | --- |
| 112225,112227 | Healthy | 0 | 10 |
|  | Lung Cancer | 10 | 0 |
|  | Liver Cancer | 3 | 7 |
| 112221,112227 | Healthy | 0 | 10 |
|  | Lung Cancer | 10 | 0 |
|  | Liver Cancer | 2 | 8 |
| 109782,109779 | Healthy | 3 | 7 |
|  | Lung Cancer | 10 | 0 |
|  | Liver Cancer | 1 | 9 |
| 207578,207570 | Healthy | 2 | 8 |
|  | Lung Cancer | 0 | 10 |
|  | Liver Cancer | 10 | 0 |
| 111158,111156 | Healthy | 10 | 0 |
|  | Lung Cancer | 7 | 3 |
|  | Liver Cancer | 0 | 10 |
| 196388,196386 | Healthy | 5 | 5 |
|  | Lung Cancer | 10 | 0 |
|  | Liver Cancer | 0 | 10 |
| 45485,45488 | Healthy | 8 | 2 |
|  | Lung Cancer | 2 | 8 |
|  | Liver Cancer | 10 | 0 |
| 73316,73308 | Healthy | 10 | 0 |
|  | Lung Cancer | 0 | 10 |
|  | Liver Cancer | 2 | 8 |
| 18865,18859 | Healthy | 0 | 10 |
|  | Lung Cancer | 10 | 0 |
|  | Liver Cancer | 4 | 6 |

* A pattern contains two or more window IDs sorted by NRs. The window with ID listed on the left always own more NRs than the one with ID on the right.
